# Echocardiographic estimation of pulmonary artery wedge pressure: invasive derivation, validation, and prognostic association beyond diastolic dysfunction grading

**DOI:** 10.1093/ehjci/jead301

**Published:** 2023-11-09

**Authors:** Thomas Lindow, Aristomenis Manouras, Per Lindqvist, Daniel Manna, Björn Wieslander, Rebecca Kozor, Geoff Strange, David Playford, Martin Ugander

**Affiliations:** Clinical Physiology, Clinical Sciences, Lund University, Lund, Sweden; Department of Clinical Physiology, Växjö Central Hospital, Växjö, Sweden; Department of Research and Development, Region Kronoberg, Växjö, Sweden; Kolling Institute, Royal North Shore Hospital, University of Sydney, Kolling Building, St Leonards, Sydney, New South Wales 2065, Australia; Department of Cardiology, Karolinska University Hospital, Karolinska Institutet, Stockholm, Sweden; Department of Clinical Physiology, Surgical and Perioperative sciences, Umeå University, Umeå, Sweden; Department of Clinical Physiology, Växjö Central Hospital, Växjö, Sweden; Department of Research and Development, Region Kronoberg, Växjö, Sweden; Department of Clinical Physiology, Växjö Central Hospital, Växjö, Sweden; Department of Clinical Physiology, Karolinska University Hospital, Karolinska Institutet, Stockholm 17176, Sweden; Kolling Institute, Royal North Shore Hospital, University of Sydney, Kolling Building, St Leonards, Sydney, New South Wales 2065, Australia; Institute for Health Research, School of Medicine, University of Notre Dame, Fremantle, Australia; Faculty of Medicine and Health, University of Sydney, Sydney, Australia; Heart Research Institute, Sydney, Australia; Royal Prince Alfred Hospital, Sydney, Australia; Institute for Health Research, School of Medicine, University of Notre Dame, Fremantle, Australia; Kolling Institute, Royal North Shore Hospital, University of Sydney, Kolling Building, St Leonards, Sydney, New South Wales 2065, Australia; Department of Clinical Physiology, Karolinska University Hospital, Karolinska Institutet, Stockholm 17176, Sweden

**Keywords:** echocardiography, heart failure, diastolic dysfunction, pulmonary capillary wedge pressure

## Abstract

**Aims:**

Grading of diastolic function can be useful, but indeterminate classifications are common. We aimed to invasively derive and validate a quantitative echocardiographic estimation of pulmonary artery wedge pressure (PAWP) and to compare its prognostic performance to diastolic dysfunction grading.

**Methods and results:**

Echocardiographic measures were used to derive an estimated PAWP (ePAWP) using multivariable linear regression in patients undergoing right heart catheterization (RHC). Prognostic associations were analysed in the National Echocardiography Database of Australia (NEDA). In patients who had undergone both RHC and echocardiography within 2 h (*n* = 90), ePAWP was derived using left atrial volume index, mitral peak early velocity (E), and pulmonary vein systolic velocity (S). In a separate external validation cohort (*n* = 53, simultaneous echocardiography and RHC), ePAWP showed good agreement with invasive PAWP (mean ± standard deviation difference 0.5 ± 5.0 mmHg) and good diagnostic accuracy for estimating PAWP >15 mmHg [area under the curve (95% confidence interval) 0.94 (0.88–1.00)]. Among patients in NEDA [*n* = 38,856, median (interquartile range) follow-up 4.8 (2.3–8.0) years, 2756 cardiovascular deaths], ePAWP was associated with cardiovascular death even after adjustment for age, sex, and diastolic dysfunction grading [hazard ratio (HR) 1.08 (1.07–1.09) per mmHg] and provided incremental prognostic information to diastolic dysfunction grading (improved *C*-statistic from 0.65 to 0.68, *P* < 0.001). Increased ePAWP was associated with worse prognosis across all grades of diastolic function [HR normal, 1.07 (1.06–1.09); indeterminate, 1.08 (1.07–1.09); abnormal, 1.08 (1.07–1.09), *P* < 0.001 for all].

**Conclusion:**

Echocardiographic ePAWP is an easily acquired continuous variable with good accuracy that associates with prognosis beyond diastolic dysfunction grading.

## Graphical Abstract

**Graphical Abstract:**
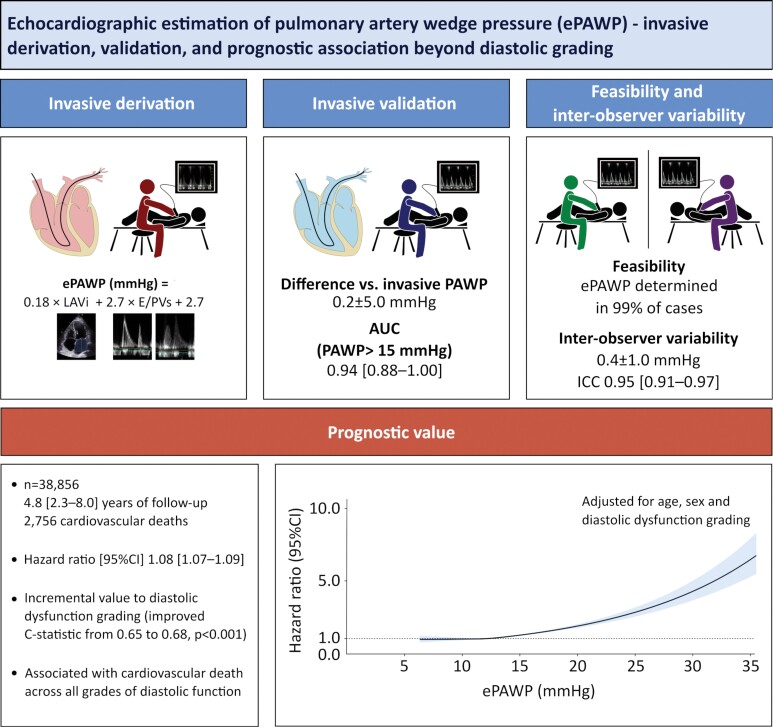
A quantitative echocardiographic estimation of pulmonary artery wedge pressure. ePAWP was derived using left atrial volume indexed to body surface area (LAVi) and mitral peak early velocity (E)/pulmonary vein peak systolic velocity (PVs), showed excellent performance in an independent validation cohort, had excellent feasibility and interobserver variability, and showed an association with prognosis in a large clinical cohort.

## Background

Elevated left ventricular filling pressures (LVFP) is an integral part of heart failure (HF) pathophysiology and diagnosis.^1^ While it is most accurately determined by invasive procedures such as left or right heart catheterization (RHC),^2,3^ echocardiography is the most commonly used method given that it is non-invasive and widely available.^4^ An accurate echocardiographic diagnosis of increased LVFP is important to establish a HF diagnosis but also to quantitatively monitor treatment effects and disease progression. In 2016, the European Association of Cardiovascular Imaging (EACVI) and the American Society of Echocardiography (ASE) provided updated guidelines on the echocardiographic assessment of diastolic function and LVFP.^5^ In these guidelines, a simplified approach is recommended in which diastolic function and LVFP are assessed through grading algorithms and discrete cut-off values for selected echocardiographic parameters associated with diastolic function and LVFP. Discrepant results have been presented regarding the accuracy for using this algorithm to detect elevated pulmonary artery wedge pressures (PAWP).^6,7^ Furthermore, an algorithmic approach to classifying into discrete grades of diastolic dysfunction in this manner has been shown to commonly lead to indeterminate classifications.^8–10^ Instead, a quantitative non-invasive estimate of LVFP as a continuous variable would be beneficial. Ideally, such an estimate should provide both an accurate and precise estimate compared with an invasive reference standard (PAWP), be easily obtained by echocardiography, have low interrater variability, and be associated with increased risk of cardiovascular events with increasing values. Therefore, we aimed to invasively derive and validate a quantitative echocardiographic estimation of PAWP, evaluate its feasibility and interrater variability, and determine its prognostic value in relation to the EACVI/ASE guidelines for grading of diastolic dysfunction.

## Methods

Four different data sets were included in this study for the distinct purposes of (i) derivation of an estimated PAWP (ePAWP) based on echocardiographic parameters, (ii) validation of ePAWP in an independent cohort, (iii) assessment of feasibility and interobserver variability of ePAWP, and (iv) the prognostic performance of ePAWP. Ethical approvals were obtained from the Human Research Ethics Committees for each cohort, respectively.

Derivation of ePAWP was performed through retrospective analysis of a cohort of consecutive patients with a clinical referral for RHC at Karolinska University Hospital, Sweden (*n* = 153), due to unclear cause of dyspnoea, advanced heart failure, suspected pulmonary arterial hypertension, or follow-up for verified pulmonary arterial hypertension. RHC was performed using a Swan–Ganz thermodilution catheter inserted through the right internal jugular vein, a medial cubital vein, or the right femoral vein. PAWP was defined as the mean PAWP and recorded at end-expirium during spontaneous breathing. Patients with at least moderate mitral valve regurgitation or non-sinus rhythms were excluded. External validation of ePAWP was performed in a separate cohort of patients who had undergone clinically indicated RHC at Umeå University Hospital, Sweden, between 2010 and 2015 (*n* = 154) with similar indications as listed above for the validation cohort. The main and/or contributing diagnoses are presented in Supplementary data online, *Tables S1* and *S2*.^11^

A comprehensive transthoracic echocardiographic exam was performed within 2 h of RHC in all patients in the derivation cohort and simultaneously with the RHC in the validation cohort. Echocardiograms were obtained using a Vivid E9 system (GE Medical Systems, Horten, Norway). Off-line analyses were done using commercially available image analysis software (EchoPAC, General Electric, Waukesha, Wisconsin, USA). Echocardiograms were digitally stored and re-analysed by an experienced operator blinded to the result of the RHC. Pulsed-wave Doppler velocities [mitral E- and A-wave, pulmonary venous systolic (PVs) and diastolic (PVd)], tissue Doppler myocardial velocities (septal and lateral e′), left atrial (LA) end-systolic volume indexed to body surface area (LAVi; Simpson biplane method), left ventricular ejection fraction (LVEF), and left ventricular mass were measured according to standard echocardiographic methods.^12^ In addition to conventional ratios of echocardiographic parameters often included in the assessment of diastolic function (E/e′, E/A, PVs/PVd), the ratio E/[(PVs + PVd)/2] was included, based on a recent observation of a close correlation with invasive PAWP using cardiovascular magnetic resonance imaging.^13^ Since PVd and mitral E are closely related,^14^ PVd could possibly be redundant, and E/PVs was therefore also included in the analysis. Tricuspid regurgitation velocity was purposely not included because of the high prevalence of absent or non-reliable spectral signals of tricuspid regurgitation during routine echocardiography.^15^ Also, the magnitude of pulmonary artery pressure, for which tricuspid regurgitant jet velocity is a surrogate measure, is affected by pulmonary vascular resistance (PVR). Elevated PAWP can be present in both isolated post-capillary hypertension and in mixed pre- and post-capillary hypertension.^16^ Including a coefficient for tricuspid regurgitation could result in severely different ePAWP based on differences in PVR. Furthermore, a sensitivity analysis was performed in which the tricuspid regurgitation was added to the final derivation model, but *R*^2^ was not improved (data not shown).

Feasibility and interobserver variability of ePAWP was assessed in a separate retrospectively identified data set consisting of 70 patients with moderate aortic stenosis who had undergone an echocardiographic examination at Växjö Central Hospital, Sweden, in 2017 as a part of clinical referral.

Prognostic performance of ePAWP was assessed in the National Echo Database of Australia (NEDA), which is a large, observational registry including individual echocardiographic data from participating centres throughout Australia.^15^ Currently, >600 000 subjects are included in the registry. Typically, included subjects have been referred by a primary care physician for echocardiography in the investigation of known or suspected heart disease. By the structure of the Australian healthcare system, minimal referral bias applies. Data have been cross-linked to the Australian National Death Index^17^ to obtain survival status for each subject until study census date (21 May 2019). In consistency with previous NEDA analyses,^8,18–20^ causes of death were categorized according to the International Classification of Diseases, Tenth Edition (ICD-10) and a primary code within I.00–I.99 was considered as a cardiovascular-related death, and the accuracy of this classification has been validated.^17^ The NEDA database has been registered in the Australian New Zeeland Clinical Trials Registry (ACTRN12617001387314).

For this study, only the last echocardiogram for each eligible subject in NEDA was included. In addition, patients with biological or mechanical mitral valve prosthesis, pacemaker, mitral stenosis or at least moderate mitral regurgitation, age <18 years, absent follow-up data, or missing values required for ePAWP or to define diastolic dysfunction grading were excluded. In addition, cases with unphysiological values (pulmonary venous or mitral velocities <0.005 m/s, LAVi <5 mL/m^2^) were excluded.

ePAWP determined using the derivation cohort was applied to all patients in the validation cohort, the feasibility cohort, and the prognostic cohort. Also, the ASE/EACVI algorithms^6^ for diastolic dysfunction (Algorithm 1) and left atrial pressure (LAP; Algorithm 2) were applied to all patients. When assessing the diagnostic and prognostic value of ePAWP using these algorithms, patients were classified based on the presence of reduced/normal LVEF as recommended.^6^ Pragmatically, patients with normal diastolic function (ASE/EACVI Algorithm 1; LVEF ≥50%) and patients with estimated normal LAP (ASE/EACVI Algorithm 2; LVEF <50%) were both classified as having normal result of the diastolic dysfunction grading. Those with indeterminate results were classified as having indeterminate diastolic function, and those with abnormal results from either algorithm were classified as having abnormal diastolic function. Of note, among patients with reduced LVEF, the ASE/EACVI algorithm does not allow for separation of patients with normal LAP and Grade I diastolic dysfunction. Because of this, when comparing the diagnostic and prognostic value of ePAWP and diastolic dysfunction grading, results are presented for the full cohort, but also stratified by LVEF.

In the feasibility cohort, which consisted of patients with moderate aortic stenosis, all patients were considered to have structural heart disease, and the second algorithm was applied to all patients, regardless of LVEF.

For comparison, a regression equation based on LAVi and mitral E only (ePAWP_E_) was also calculated, as well as two previously described echocardiographic PAWP estimations.^21,22^ The equation from Pozzoli *et al.*^21^ included information on mitral E, deceleration time, and PVs/(PVs + PVd) [estimated PAWP = 1.85 × mitral E/mitral E-wave deceleration time (m/s^2^) − 0.1 × PVs/(PVs + PVd) (%) + 10.9], while the equation from Nagueh *et al.*^22^ included only E/e′ (estimated PAWP = 1.24 × E/e′ + 1.9).^22^ All estimates of PAWP were tested for agreement with invasive PAWP in the validation data set. In addition, a subset of the prognostic data set was selected, in which only cases with all parameters required for all estimates of PAWP were present.

### Statistical analysis

ePAWP was derived by first applying univariable linear regression to echocardiographic parameters that were presumed to be related to PAWP (LAVi, mitral A, mitral E, PVs, PVd, e′, LV mass, LVEF) including relevant variable ratios [E/A, E/e′, PVs/PVd, E/(PVs + PVd), E/PVs]. In a stepwise selection of variables, multivariable linear regression analysis was then performed by including those with the highest *R*^2^ in the univariable analysis. Variables were retained in the model only if the adjusted *R*^2^ increased after addition of a new variable. The variance inflation factor (VIF) was determined for the variables included in the final model to assess multi-collinearity, with low multi-collinearity pre-specified as VIF <3.

The relationship between ePAWP and invasively measured PAWP was described using scatter- and Bland–Altman plots in the derivation and validation cohorts, respectively. Diagnostic performance of ePAWP was evaluated using receiver operating characteristics (ROC) analysis for the detection of elevated PAWP, defined as >15 mmHg,^3^ and results are presented as the area under the curve (AUC) [95% confidence interval (CI)]. The *F*-test was used to test for difference in the size of standard deviations as measures of precision.

When diagnostic accuracy of ePAWP was assessed, both the derivation and validation cohorts were combined, and results are presented as sensitivity, specificity, positive predictive value, negative predictive value, positive likelihood ratio (PLR), and inverse negative likelihood ratio (INLR). Comparison of the proportions of correct classifications when discrepant results were obtained by ePAWP and diastolic dysfunction grading was performed with the χ^2^ test. Diagnostic accuracy for ePAWP among those with indeterminate diastolic classification is presented separately.

In order to evaluate whether the diagnostic performance of ePAWP depended on LVEF, a sensitivity analysis was performed in patients with LVEF <50% and ≥50% separately.

Feasibility was calculated as the number of examinations with successfully determined ePAWP. Interobserver variability was described as the mean ± SD difference between the two observers and intraclass correlation (ICC) (95% CI). The association between ePAWP and cardiovascular death was evaluated using Cox regression analysis both unadjusted and adjusted for age, sex, and diastolic dysfunction grade. In addition, to account for the effect of competing risk of death on the analysis using cardiovascular death as an outcome, competing risks regression as described by Fine and Gray was applied and presented with subhazard ratios (SHR) with 95% CI. Prognostic performance was described with the *C*-statistic. Differences in *C*-statistics between diastolic dysfunction grading and ePAWP were evaluated using Harrell’s *C*-index. The incremental value of ePAWP beyond diastolic dysfunction grading was assessed both by describing the increase in the *C*-statistic after addition of ePAWP to diastolic dysfunction grading and after stratifying patients based on diastolic dysfunction grade. Model improvement was assessed using the likelihood ratio test. The impact of increasing ePAWP was further described as a hazard ratio (95% CI) for cardiovascular mortality using Cox regression modelled with natural cubic splines with three knots (25th, 50th, and 75th percentiles), unadjusted and adjusted for age, sex, and diastolic dysfunction grading. Statistical significance was accepted at the level of *P* < 0.05 (two-sided). Statistical analysis was performed using R version 4.2.1 (R Core Team, Vienna, Austria, example packages: Survival v. 3.4-0,^23^ pROC v. 1.18.0,^24^ and rms v. 6-3-0).^25^

## Results

### Derivation and validation of accuracy for ePAWP

In the derivation cohort, one patient with missing PAWP, 71 patients with non-sinus rhythm, 27 patients with at least moderate mitral regurgitation, 2 patients with mitral stenosis, and 2 patients with missing pulmonary vein velocities were excluded, resulting in 90 patients to be finally included. Baseline characteristics are presented in *Table 1*. LAVi and mitral E/PVs were the two variables that were most strongly associated with PAWP in univariable linear regression, and both were included in a multivariable model. Since no other variable provided incremental value to the multivariable model, these were the only variables finally retained (*Table 2*). This resulted in the regression equation ePAWP = 0.179 × LAVi + 2.672 × mitral E/PVs + 2.7, in which ePAWP is given in mmHg, LAVi in mL/m^2^, and mitral E and PVs in the same units of velocity (e.g. both in m/s). A regression equation based on LAVi and mitral E only (ePAWP_E_) was also calculated and is presented as Supplementary data online, Material.

**Table 1:** Baseline clinical and echocardiographic characteristics

	Derivation cohort	Validation cohort
*n*	90	53
Age, years	59.6 ± 16.6	59.8 ± 13.2
Male sex, *n* (%)	46 (51)	28 (28)
Height, cm	170 ± 11	167 ± 8
Weight, kg	79 ± 20	72 ± 16
BMI, kg/m^2^	27.1 ± 5.8	25.7 ± 4.8
BSA, m^2^	1.89 ± 0.26	1.82 ± 0.25
Diabetes, *n* (%)	14 (16)	5 (9)
Hypertension, *n* (%)	55 (61)	19 (35)
Hypercholesterolaemia, *n* (%)	27 (30)	—
Current/former smoker, *n* (%)	25 (28)	—
NYHA class, *n* (%)		—
2	11 (18)	—
3	39 (63)	—
4	4 (7)	—
NT-proBNP, ng/L	561 [307–2228]	518 [189–1657]
Hb, g/L	130 ± 21	—
Creatinine, µmol/L	90 ± 35	—
Systolic blood pressure, mmHg	124 ± 21	132 ± 20
Diastolic blood pressure, mmHg	69 ± 13	77 ± 9
PAWP, mmHg	15.3 ± 7.7	12.9 ± 6.7
mPAP, mmHg	25.9 ± 10	30.8 ± 12.9
PVR, WU	2.3 ± 1.6	4.0 ± 3.3
LVEDVi, mL/m^2^	60 ± 28	51 ± 26
LAVi, mL/m^2^	39 ± 15	29 ± 14
LVMi, g/m^2^	98 ± 35	87 ± 37
LVEF, %	57 ± 20	54 ± 14
Mitral E, m/s	0.88 ± 0.28	0.79 ± 0.24
Mitral A, m/s	0.67 ± 0.34	0.66 ± 0.23
E/A, unitless	1.80 ± 1.46	1.41 ± 0.88
PVs, m/s	0.50 ± 0.19	0.46 ± 0.15
PVd, m/s	0.60 ± 0.22	0.46 ± 0.16
E/e′	13 ± 5	11 ± 6
SPAP, mmHg	43 ± 14	52 ± 20

Data are presented as mean ± standard deviation or median (interquartile range) (NT-proBNP), unless stated otherwise.

BMI, body mass index; BSA, body surface area; LAVi, left atrial volume indexed to body surface area; LVEDVi, left ventricular end-diastolic volume indexed to body surface area; LVEF, left ventricular ejection fraction; LVMi, left ventricular mass indexed to body surface area; NT-proBNP, N-terminal pro-B-type natriuretic peptide; NYHA class, New York Heart Association functional classification; SPAP, systolic pulmonary artery pressure.

**Table 2:** Results of the uni- and multivariable regression analyses for prediction of invasive pulmonary artery wedge pressure using echocardiography

	Univariable analysis		Multivariable model^a^			
	*R* ^2^	*P*	*B* coefficient	*t*	*P*	VIF
LAVi	0.24	<0.001	0.179	4.94	<0.001	1.11
E/PVs	0.22	<0.001	2.672	4.96	<0.001	
PVs/PVd	0.21	<0.001				
E	0.19	<0.001				
PVd	0.17	<0.001				
E/A	0.11	0.002				
E/(PVs + PVd/2)	0.10	0.004				
PVs	0.09	0.003				
E/e′	0.05	0.04				
LVM	0.02	0.002				
LVEF	0.02	0.01				
A	0.02	0.23				
e′	0.02	0.28				

A, mitral A-wave velocity (m/s); E, mitral E-wave velocity m/s; LAVi, left atrial volume indexed to body mass index; LVEF, left ventricular ejection fraction (%); LVM, left ventricular mass (g); PVd, pulmonary vein diastolic velocity (m/s); PVs, pulmonary vein systolic velocity (m/s); VIF, variance inflation factor.

^a^Intercept: 2.70. *R*^2^ of the final model: 0.48, *P* < 0.001. Adding additional variables to the multivariable prediction model did not improve the adjusted *R*^2^.

**Figure 1:**
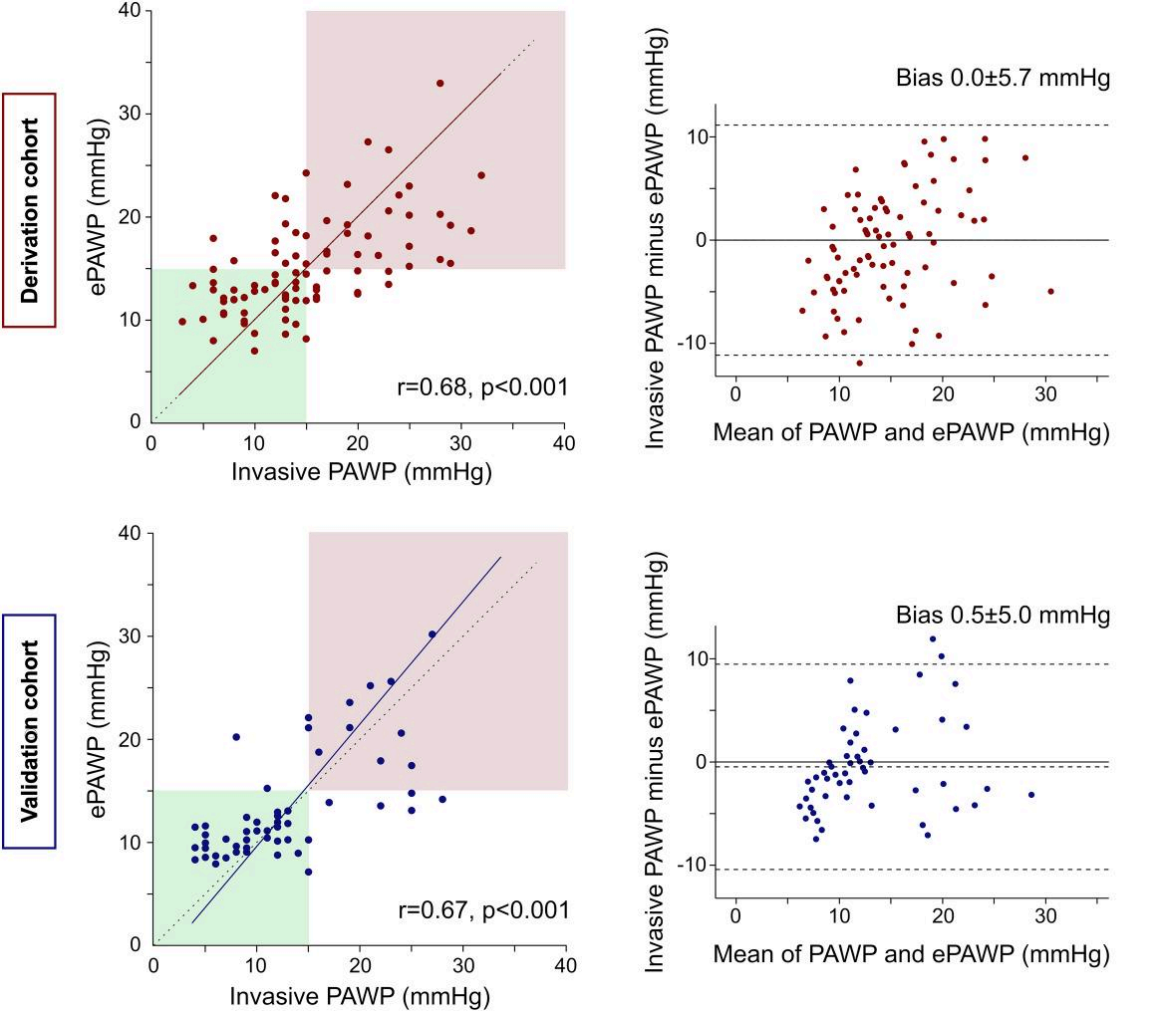
Derivation and validation of ePAWP. *Left panels:* Scatterplots showing the relation between pulmonary artery wedge pressure (PAWP) as assessed by echocardiography (ePAWP) and according to right heart catheterization (invasive PAWP). The upper right red and lower left green boxes denote correct classifications of lower (lower left green) and high (upper right red) invasively measured PAWP defined as ≤ or >15 mmHg, respectively. Upper left panel: derivation cohort. Lower left panel: validation cohort. *Right panels:* Bland–Altman plots showing the difference between ePAWP and invasively measured PAWP vs. the mean of invasive PAWP and ePAWP in the derivation (upper panel) and validation (lower panel) cohorts.

In the validation cohort, 13 patients were excluded due to missing PAWP, 32 patients due to a non-sinus rhythm, and 56 patients due to missing values needed for ePAWP estimation (LAVi, *n* = 5; mitral E, *n* = 4; PVs, *n* = 47). Among the remaining 53 patients, ePAWP showed good agreement with *invasive* PAWP (mean ± SD difference 0.5 ± 5.0 mmHg). The relationship between ePAWP and PAWP is presented for both the derivation and validation cohort in *Figure 1*.

In the validation cohort, ePAWP showed excellent diagnostic accuracy for estimating PAWP >15 mmHg [AUC 0.94 (0.88–1.00)]. After combining both the derivation and validation cohort (*n* = 143), the optimal cut-off was 14.6 mmHg [sensitivity, 78% (63–88); specificity, 81% (71–88); PLR, 4.1 (2.6–6.3); INLR, 3.6 (2.1–6.3)]. Sensitivity, specificity, positive and negative predictive values, accuracy, and likelihood ratios are presented for different ePAWP cut-offs in *Table 3*.

**Table 3:** Sensitivity, specificity, positive and negative predictive values, accuracy, and likelihood ratios on different ePAWP cut-offs for detection of elevated pulmonary arterial wedge pressure in the combined derivation and validation cohorts (*n* = 143)

ePAWP (mmHg)	Sensitivity	Specificity	Accuracy	PPV	NPV	PLR	INLR
12.0	100	56	71	54	100	2.3	—
12.5	96	63	74	57	97	2.6	15.4
13.1	90	71	78	62	93	3.1	7.0
13.5	84	75	78	63	90	3.3	4.6
13.8	82	78	79	66	89	3.7	4.2
14.6	78	81	80	68	87	4.0	3.6
15.1	69	82	78	67	84	3.8	2.7
15.5	67	84	78	69	83	4.2	2.6
15.8	65	86	79	71	83	4.7	2.6
16.2	63	87	79	72	82	5.0	2.4
16.6	57	88	78	72	80	4.9	2.1
17.8	51	89	76	71	78	4.8	1.8
18.0	49	90	76	73	77	5.1	1.8
18.6	45	93	76	76	76	6.0	1.7
19.5	37	94	74	75	77	5.8	1.5
20.2	33	95	73	76	76	6.1	1.4

INLR, inverse negative likelihood ratio; NPV, negative predictive value; PLR, positive likelihood ratio; PPV, positive predictive value.

After excluding patients with indeterminate diastolic function (*n* = 28), sensitivity for correctly classifying invasive PAWP findings for diastolic dysfunction grading was lower 65% (48–79) than for ePAWP [78% (62–89)], as was specificity [diastolic dysfunction grading, 76% (65–85); ePAWP, 84 (74–91)]. In patients with indeterminate diastolic dysfunction grading [*n* = 28 (20%)], ePAWP correctly identified a low/normal PAWP in 71% of cases and an elevated PAWP in 78% of cases. Discrepant classifications between ePAWP and diastolic dysfunction grading occurred in 27 cases (24%). Among these, ePAWP correctly classified invasive PAWP findings in 70% of cases and diastolic dysfunction grading in 30% (*P* < 0.001).

After splitting the derivation and validation cohorts by LVEF for the purpose of sensitivity analysis, the AUC for the detection of elevated PAWP was 0.82 (0.70–0.95) for patients with LVEF <50% (*n* = 42) and 0.87 (0.80–0.94) for patients with LVEF ≥50% (*n* = 101).

### Feasibility and interrater variability

The feasibility cohort consisted of 70 patients with moderate aortic stenosis (mean age 76.7 ± 10.5 years, 66% males). A Grade II/III diastolic dysfunction was present in 35 patients (50%) and normal LAP/Grade I diastolic dysfunction in 26 (37%), and the remaining 9 (13%) cases were indeterminate. ePAWP could be determined in 69 out 70 (99%) of patients. Interrater variability was excellent [mean ± SD difference 0.4 ± 1.0 mmHg, ICC 0.95 (0.91–0.97)].

### Prognostic value of ePAWP

**Figure 2:**
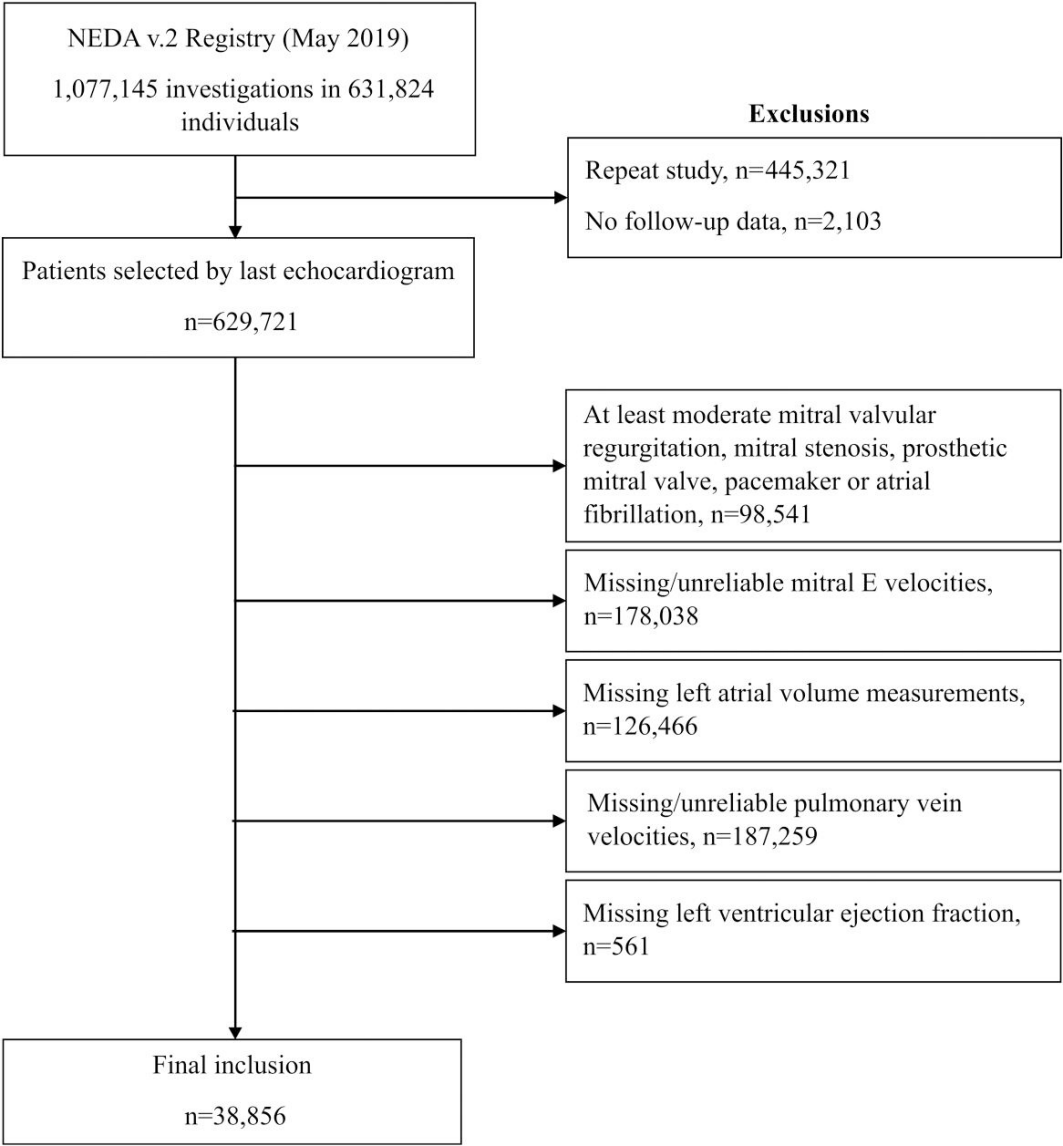
Study flowchart. Flowchart of patient inclusion and exclusion in the prognostic data set (NEDA).

In the prognostic cohort, ePAWP was applied to 38 856 patients (mean age 58.2 ± 17.6 years, 53% males) with a median (interquartile range) follow-up of 4.8 (2.3–8.0) years. A flowchart of patient inclusion and exclusion in this data set is presented in *Figure 2*. A reduced LVEF was found in 3357 (8.5%), among which 770 (23%) were classified as having elevated LVFP according to the ASE/EACVI algorithm, 1660 (49%) had normal LVFP, and 921 (27%) cases were indeterminate. In patients with LVEF ≥50%, diastolic dysfunction grading resulted in 28 569 (81%) patients with normal diastolic function, 4666 (13%) with indeterminate diastolic function, and 2264 (6.4%) patients with abnormal diastolic function. In summary, in the combined group of patients with both reduced and preserved LVEF, 30 235 (78%) were classified as normal, 5587 (14%) as indeterminate, and 3034 (7.8%) as abnormal according to the diastolic classification.

**Figure 3:**
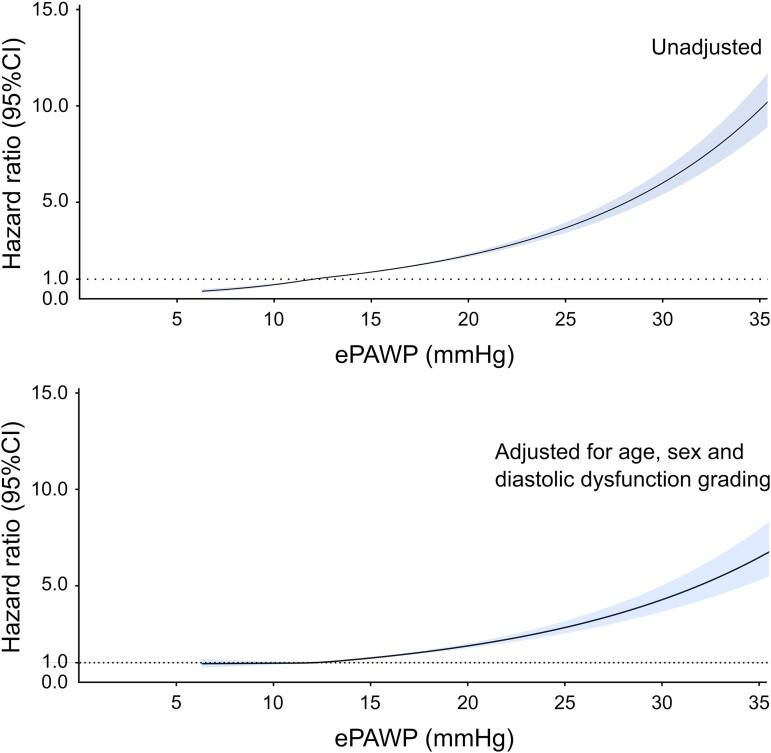
Impact of increasing ePAWP on the risk of cardiovascular death. The hazard ratio [with 95% confidence intervals (CI)] for cardiovascular mortality was calculated using Cox regression and modelled with natural cubic splines with three knots (25th, 50th, and 75th percentiles), unadjusted (upper panel), and adjusted for age, sex, and diastolic dysfunction grading (lower panel).

During follow-up, 2756 (7.1%) cardiovascular deaths occurred. ePAWP was associated with cardiovascular death even after adjustment for age, sex, and diastolic classification [unadjusted HR 1.11 (1.10–1.11) per mmHg; adjusted HR 1.08 (1.07–1.09)] and with similar results after accounting for competing risks [unadjusted SHR 1.11 (1.09–1.11) per mmHg; adjusted HR 1.07 (1.06–1.08)]. C-statistics did not differ for ePAWP compared with diastolic dysfunction grading [0.64 (0.63–0.65) vs. 0.65 (0.64–0.66), *P* = 0.29], but ePAWP provided incremental prognostic information beyond diastolic dysfunction grading alone [improvement in *C*-statistics from 0.65 (0.64–0.66) to 0.68 (0.67–0.69), *P* < 0.001]. The impact of increasing ePAWP as a continuous measure on the risk for cardiovascular death is presented in *Figure 3*. ePAWP was associated with cardiovascular mortality across both sexes, among all diastolic dysfunction grades, in both reduced and preserved LVEF, and across all age groups (*Table 4*).

**Table 4:** Association between ePAWP and cardiovascular death after stratification based on diastolic dysfunction grading, left ventricular ejection fraction, and sex

	Diastolic dysfunction grade					
	Normal (*n* = 30,235, 1447 events) HR (95% CI)		Indeterminate (*n* = 5587, 754 events) HR (95% CI)		Abnormal (*n* = 3034, 555 events) HR (95% CI)	
	Unadjusted	Adjusted^a^	Unadjusted	Adjusted^a^	Unadjusted	Adjusted^a^
ePAWP (mmHg)	1.08 (1.07–1.10)	1.07 (1.06–1.09)	1.09 (1.08–1.10)	1.08 (1.07–1.09)	1.09 (1.07–1.11)	1.08 (1.07–1.09)
	**Normal LVEF** (*n* = 35,500, 2131 events)			**Reduced LVEF** (*n* = 2362, events 429)		
	HR (95% CI)			HR (95% CI)		
	Unadjusted	Adjusted^b^		Unadjusted	Adjusted^b^	
ePAWP (mmHg)	1.10 (1.10–1.11)	1.07 (1.06–1.08)		1.12 (1.10–1.13)	1.09 (1.07–1.11)	
	**Male patients** (*n* = 20,623, 1623 events)			**Female patients** (*n* = 18,233, 1333 events)		
	HR (95% CI)	HR (95% CI)		HR (95% CI)	HR (95% CI)	
	Unadjusted	Adjusted^c^		Unadjusted	Adjusted^c^	
ePAWP (mmHg)	1.11 (1.10–1.12)	1.08 (1.07–1.09)		1.11 (1.10–1.11)	1.09 (1.08–1.09)	

	Age (quartiles)							
	<46 years (*n* = 9379, 80 events)HR (95% CI)		46–60 years (*n* = 9,928, 269 events)HR (95% CI)		60–72 years (*n* = 10,221, 631 events)HR (95% CI)		>72 years (*n* = 9958, 1776 events)HR (95% CI)	
	Unadjusted	Adjusted^d^	Unadjusted	Adjusted^d^	Unadjusted	Adjusted^d^	Unadjusted	Adjusted^d^
ePAWP (mmHg)	1.10 (1.08–1.13)	1.10 (1.07–1.13)	1.11 (1.09–1.13)	1.09 (1.06–1.12)	1.09 (1.08–1.10)	1.07 (1.05–1.09)	1.10 (1.09–1.11)	1.09 (1.08–1.10)

CI, confidence intervals; HR, hazard ratio; LVEF, left ventricular ejection fraction.

^a^Adjusted for age and sex.

^b^Adjusted for age, sex, and diastolic dysfunction grading.

^c^Adjusted for age and diastolic dysfunction grading .

^d^Adjusted for sex and diastolic dysfunction grading.

### Comparison with previous ePAWP estimations

A comparative overview for ePAWP, ePAWP_E_, and PAWP as estimated by Pozzoli *et al.*^21^ and Nagueh *et al.*^22^ regarding agreement with invasive PAWP and prognostic strength is presented in *Table 5*. Compared with ePAWP for the current study, the equation by Pozzoli *et al.*^21^ resulted in worse accuracy but similar precision (mean ± SD difference 4.2 ± 5.0 mmHg, *P* < 0.001 for accuracy, *P* = 0.87 for precision) (*Table 5*). The equation by Nagueh *et al.*^22^ showed similar accuracy but lower precision (mean ± SD difference 2.7 ± 7.9 mmHg, *P* = 0.10 for accuracy, *P* = 0.002 for precision). All methods were associated with increased risk of cardiovascular death after adjustment for age, sex, and diastolic dysfunction grading, with ePAWP being associated with the greatest risk increase per mmHg. For reference, the results from another echocardiographic estimation of PAWP by Chubuchny *et al.*^26^ are also presented in *Table 5*, although it was not tested in the validation data set, or the prognostic data set, due to lack of the necessary measured echocardiographic parameters in our data.

**Table 5:** Comparative overview of available quantitative echocardiographic methods for the estimation of pulmonary artery wedge pressure (PAWP) regarding agreement with invasive reference and prognosis (cardiovascular mortality)

Method	Formula	Difference vs. invasive PAWP (mmHg)^a^	AUC (PAWP >15 mmHg)	Hazard ratio (95% CI)^b^	
				Unadjusted	Adjusted for age, sex, and diastolic dysfunction grade
ePAWP	0.179 × LAVi + 2.672 × mitral E/PVs + 2.7	0.2 ± 5.0	0.94 (0.88–1.00)	1.10 (1.10–1.11)	1.09 (1.08–1.11)
ePAWP_E_	0.230 × LAVi + 0.102 × mitral E − 2.7	−0.9 ± 5.6	0.88 (0.79–0.98)	1.08 (1.07–1.09)	1.05 (1.04–1.06)
Pozzoli^21^	1.85 × mitral E/DT − 0.1 × PVs/(PVs + PVd) + 10.9	4.2 ± 5.0	0.95 (0.90–1.00)	0.97 (0.96–0.99)	1.06 (1.05–1.08)
Nagueh^22^	1.24 × E/e′ + 1.9	2.8 ± 7.9	0.77 (0.59–0.94)	1.09 (1.08–1.09)	1.07 (1.06–1.08)
Chubuchny^26^	0.5 × LVEF − 0.1 × TRV + 0.7 E/e′ + 1.0 × RVFAC + 0.9 × IVC + 0.3 × LAVi + 43	0.7 ± 4.1^c^	0.97 (0.96–0.98)^c^	^c^	^c^

AUC, area under the curve; CI, confidence interval; LAVi, left atrial volume indexed to body surface area (mL/m^2^); mitral E, mitral early peak wave velocity (m/s); PVs, systolic peak pulmonary vein velocity; DT, deceleration time (s); LVEF, left ventricular ejection fraction (%); PVd, diastolic peak pulmonary vein velocity; PVs/(PVs + PVd) was measured in %; TRV, tricuspid regurgitation peak velocity (cm/s); RVFAC, right ventricular fractional area change (%); IVC, inferior vena cava (mm).

^a^Differences between estimates of PAWP and invasive PAWP were evaluated in the validation cohort of the current study.

^b^The prognostic value was evaluated in a subset of patients in which all parameters required for ePAWP and ePAWP_E_ and the equations from Pozzoli *et al.* and Nagueh *et al.* were available [17 233 patients, 1429 cardiovascular deaths, median follow-up 5.5 (2.5–8.4) years].

^c^The results from Chubuchny *et al.* are from their original publication^26^ and were not tested in the current validation data set or the prognostic data set due to the lack of measurement of the requisite echocardiographic measures necessary to calculate the estimate of PAWP.

## Discussion

Echocardiographic ePAWP is an easily acquired, continuous variable with good accuracy which provides incremental diagnostic and prognostic value to diastolic dysfunction grading. Compared with the reference standard of invasive PAWP assessment at RHC, ePAWP could accurately estimate the presence of increased PAWP. Currently, guidelines recommend an integrated approach to diastolic dysfunction grading, in which discrete cut-offs are applied to echocardiographic parameters (LAVi, e′, E/e′, and tricuspid regurgitation velocity).^5^ A quantitative measure of PAWP provides a possibility to better describe the full spectrum of LVFP and, possibly, to detect a change in haemodynamic status in HF patients with greater accuracy and nuance than a categorical classification of increased or normal LVFP. The ePAWP formula could easily be incorporated into standard echocardiographic acquisition protocols and added to existing analysis and reporting software for automatic calculation and immediate presentation.

ePAWP was associated with an exponentially increased risk of cardiovascular death, even after adjusting for age, sex, and diastolic dysfunction grading. Future studies on the change of ePAWP as an effect of treatment changes are needed, as well as the prognostic value of such changes. Notably, estimations of PAWP could also be made using only LAVi and E and without PVs (ePAWP_E_), but its association with outcomes was less strong compared with ePAWP (see Supplementary data online, Material). ePAWP was associated with CV mortality even after adjusting for age, sex, and diastolic dysfunction grading. However, the increase in *C*-statistic beyond diastolic dysfunction was relatively modest. A modest difference in risk prediction when comparing a diastolic grading algorithm and ePAWP is expected, since the purpose of both approaches is similar, i.e. to detect an elevated LAP. Also, similar measures are included in both, e.g. LAVi, which is known to be associated with survival.^27^ Since invasively measured PAWP is associated with mortality,^28^ a relation between the non-invasive PAWP and invasive PAWP is strengthened if an association with mortality for the non-invasive surrogate is present. Notably, the prognostic association strengthens the usefulness of both diastolic dysfunction grading and ePAWP. It should be noted that the interpretation of the magnitude of the increase in *C*-statistic is not straightforward and should not be interpreted as the sole measure of predictive strength.^29^ In this case, the HR for ePAWP even after adjusting for age, sex, and diastolic dysfunction grading suggests a statistically significant and clinically meaningful improvement in risk assessment.

Furthermore, the most clinically important additive value of ePAWP in comparison to an algorithmic approach such as diastolic dysfunction grading lies with its quantitative information as a continuous variable, its improved accuracy vs. invasive PAWP, and the absence of indeterminate classifications.

The current study found that LA volume, mitral E, and PVs are all important determinants of PAWP.^14,21,30–33^ These parameters are independently associated with LAP but can also be considered to embrace distinctive aspects of LV filling. Mitral E-wave velocities describe the pressure gradient between the LV and LA during early LV relaxation. PVs reflects, in principle, the pressure gradient between the pulmonary venous circulation and LA during ventricular systole while also reflecting LA systolic filling.^14,30,31^ By comparison, LAVi carries information on the duration and severity of increased LAP leading to LA remodelling and may also increase in the presence of atrial fibrillation.^34^ LA volume suggests a chronic increase in LAP. Indeed, increased LAVi is associated with both diastolic dysfunction and reduced survival.^32^

Including a ratio between mitral E and PVs in the estimation of PAWP can also be considered to account for the close relation between the restoring forces released after the conclusion of LV contraction and the lengthening load at mitral valve opening.^5,35^ Systolic pulmonary venous flow occurs when the mitral annular plane systolic excursion (MAPSE) increases the LA volume and consequently reduces LAP. Consequently, an easily obtained measure such as MAPSE could potentially contribute to the diagnostic and prognostic performance of ePAWP, in particular in cases with more challenging PVs acquisition.^36^ However, MAPSE measurements were not routinely performed in our study cohorts. Notably, in contrast to MAPSE, PVs is likely to a larger extent affected by for example LAP, LA chamber stiffness, and transpulmonary driving forces, which are all important factors in LAP increase. In the absence of mitral valve pathologies, decreasing PVs reflects an increased LAP and reduced LV contraction from which reduced LV recoil will follow. This relates both temporally and functionally to the LA reservoir function, which has been shown to be strongly associated with LVFP and prognosis, in particular when assessed using LA strain.^37^ In the absence of mitral valvular disease, high output states, or atrial fibrillation, an increased mitral E was expected to provide valuable information on LVFP, given the information on the pressure gradient between the LA and LV during early diastole, but it is preload dependent.^38^ Adjusting for the pressure gradient between the pulmonary venous circulation and the LA before early relaxation, represented by PVs, could potentially increase the diagnostic accuracy and precision. Nonetheless, mitral E, PVs, and LAVi are all affected by loading conditions, such as mitral valvular regurgitation or stenosis, or high output states, as well as rhythm conditions, e.g. atrial fibrillation. Thus, ePAWP was currently studied only in the absence of such conditions and should not be applied in their presence.

In this study, tricuspid regurgitant jet velocities were purposely not included, since absent or non-reliable spectral signals during routine echocardiography are common.^39^ Furthermore, although increased LAP usually is followed by an increase in pulmonary artery pressure, the magnitude may differ even in HF patients, for example, due to differences in pulmonary vascular resistance.^16^ Furthermore, a sensitivity analysis was performed in which the tricuspid regurgitation was added to the final derivation model, but *R*^2^ was not improved (data not shown).

E/e′, which has been extensively studied as a marker of elevated LVFP,^40^ did not add value to the estimation of PAWP. This is in line with other reports correlating echocardiographic measures to PAWP,^41,42^ although conflicting results exist.^22,40^ As in our study, e′ and E/e′ have both been found to be poorly correlated with PAWP, while mitral E was more closely correlated with PAWP in patients with sinus rhythm.^41^ One issue with E/e′ is that e′ is significantly associated with age,^43^ which reduces specificity of E/e′ in older age groups,^44^ among which HFpEF, in particular, is common. However, age differences may be an issue with ePAWP as well. With age, mitral E is known to decrease and PVs to increase,^45^ while LAVi is fairly constant across ages.^43^ Since low PVs is common in younger individuals, ePAWP can potentially overestimate LVFP among those. However, despite these theoretical potential shortcomings, the prognostic value of ePAWP was maintained across different age groups in the current study.

There are no indeterminate classifications using the continuous variable ePAWP provided that all required measurements (LAVi, E, PVs) have been successfully obtained. In addition to the absence of indeterminate classifications, ePAWP was more often correct than diastolic dysfunction grading in the detection of elevated filling pressures. Also, sensitivity and specificity were numerically higher than for the ASE/EACVI algorithms. More importantly, the quantitative nature of ePAWP has a larger diagnostic potential than algorithms with binary outcomes, since this allows for a greater possibility to describe the full spectrum of LV filling pressures and possibly to detect a change in the haemodynamic status in HF patients with greater nuance than a categorical classification. Also, its diagnostic accuracy in detection of elevated PAWP can be described along a continuous spectrum of cut-offs with different sensitivity and specificity, allowing for interpretation of the results to be adjusted in relation to pre-test probabilities. Of note, when comparing the diagnostic accuracy between diastolic dysfunction grading and ePAWP, it should be acknowledged that diastolic dysfunction does not equate to LV filling pressures, although these concepts are intertwined and their assessment include similar echocardiographic measures.

In contrast to previously published echocardiographic estimations of PAWP from Pozzoli *et al.*,^21,22^ ePAWP from the current study offers a favourable combination of excellent mean accuracy, acceptable precision, and strength of association with outcomes. Pozzoli *et al.* provided an equation for non-invasive estimations of PAWP in patients with dilated cardiomyopathy, which included mitral flow deceleration rate (mitral E/deceleration time) and PVs/(PVs + PVd).^21^ When that equation was applied to our data, precision was similar but accuracy was markedly lower. Also, that estimate of PAWP required the measurement of E-wave deceleration time, which is known to be limited by poor reproducibility.^46^ The equation from Nagueh *et al.*^22^ was based on E/e′ and was inferior regarding precision for estimating PAWP. While all three methods were associated with increased risk of cardiovascular death, ePAWP was associated with the greatest risk increase per mmHg.

Although accuracy of ePAWP was excellent, precision was somewhat limited, albeit still with clinically useful performance, which should be interpreted in the light of variability between repeated invasively measured PAWP (0.5 ± 2.1 mmHg).^47^ This suggests that ePAWP is likely an excellent non-invasive measure to be used as a surrogate for PAWP in larger studies but may have more limited diagnostic performance in individual patients. However, when using a binary cut-off, discriminatory ability was excellent. Future studies in larger and diverse populations are justified to determine the ability of ePAWP in individual patients to determine the progression of disease or response to treatment. This would enable the ability to reap the benefits of a quantitative estimate of PAWP despite its limitations in precision compared with invasive PAWP.

In contrast to the simplicity of ePAWP, Chubuchny *et al.* obtained improved precision and higher diagnostic accuracy with a complex approach which necessitates measurement of tricuspid regurgitation velocities, LVEF, right ventricular fractional area change, LAVi, E/e′, diameter of the inferior vena cava, estimated right atrial pressure, and pulmonary regurgitation end-diastolic gradients.^26^ That approach is arguably less attractive due to it necessitating multiple measurements that may not be routinely measured and the limited ability to quantify the tricuspid regurgitant velocity in many patients.^39^

Relating the isovolumetric relaxation time (IVRT) to the time difference between E and e′ (*T*_E−e′_) has been shown to accurately detect elevated PAWP, even in patients with mitral valve regurgitation.^48^ IVRT/*T*_E−e′_ was not included in our data set, and a direct comparison of these methods has not been performed. IVRT is affected by heart rate and arterial pressure, and the acquisition of *T*_E−e′_ is dependent on detailed sampling and identification of identical RR intervals and thus may be less attractive to use in clinical routine.^5^

### Limitations

Due to the relatively large numbers of patients in the validation cohort being excluded due to missing data, the final sample was small, and further external validation is needed before clinical implementation. Pulmonary vein velocities have not been included in the recent guidelines of diastolic dysfunction grading, and recording of these measures has therefore likely become rarer and to some extent based on local routines and tradition. In centres in which pulmonary vein velocities are still being recorded routinely, such as in the feasibility cohort, adequate recordings could be obtained in the nearly every case, with excellent interobserver variability. This is consistent with previous reports of high feasibility of pulmonary vein velocity acquisitions.^49^ ePAWP_E_, which does not require PVs, is an acceptable alternative with similar accuracy and precision as ePAWP, albeit with less strong association with mortality.

Although interrater variability was excellent when measurements were performed on the same set of images, variability due to differences in acquisition needs to be determined.

Similar to the ASE/EACVI recommendations on diastolic dysfunction grading,^5^ ePAWP was not applied to patients in atrial fibrillation or in patients with at least moderate mitral valvular lesions. Thus, ePAWP does not exclude additional patients from being evaluated, but future studies should explore the possibility to derive a quantitative PAWP estimation in such patients as well.

A potential sex difference in PAWP estimation based on echocardiography was not evaluated in this study. The study cohorts are small, and it is likely that other differences between the male and female subgroups, such as differences in pulmonary hypertension profile, age, or heart failure characteristics, would confound a difference based on sex alone (see Supplementary data online, *Table S3*). That said, the performance of ePAWP remained associated with prognosis despite adjustment for sex. Future studies may determine the need of sex-specific estimations.

The data in the prognostic data set stem from an echocardiographic registry and echocardiograms performed during clinical routine. Detailed quality assessment of the individual recorded values cannot be made. To account for this, we excluded cases with non-physiological values, assumed to be erroneous. The prognostic evaluation is further limited by the lack of data on comorbidities. Although this may affect the interpretation of ePAWP as an independent predictor of cardiovascular mortality, it likely has only minor effect on determining the potential incremental value of ePAWP to diastolic dysfunction grading, as well as on the comparison with other quantitative measures.

Applying a regression equation in clinical practice typically requires that calculations are performed automatically. This can easily be achieved by including the ePAWP formula in existing echocardiographic analysis or reporting software.

Patients with atrial fibrillation or pacemaker rhythm were excluded from the study, and it is therefore not known how ePAWP performs in patients with non-sinus rhythms. Estimation of LVP in AF is complicated, and the algorithmic EACVI/ASE approach is neither recommended to be used in AF.^5^ Similarly, both quantitative estimations of PAWP by Nagueh *et al.* and Pozzoli *et al.* were studied in data sets where patients in non-sinus rhythms were excluded. Nonetheless, patients with AF constitute a significant number of patients with known or suspected HF,^50^ and future studies to improve detection and quantification of LVP in patients with AF are needed.

The derivation of ePAWP was performed by including echocardiographic parameters that were available in the data set. Diagnostic and prognostic value could perhaps be improved by incorporating LA strain parameters, which have been shown to be both diagnostically and prognostically relevant.^37^ However, LA volume and its increase during systole are important determinants of LA reservoir strain,^36^ both of which are to some extent included in ePAWP (LAVi and PVs). A quantitative measure of PAWP would be useful to determine symptom progression and relief. However, it is not known whether changes in LA volume occur rapidly enough to reflect changes in LVFP as determined by ePAWP and future studies are needed to determine this.

## Conclusion

Echocardiographic ePAWP is an easily acquired continuous variable with good accuracy that is associated with prognosis beyond diastolic dysfunction grading.

## Supplementary data

Supplementary data are available at *European Heart Journal - Cardiovascular Imaging* online.

## Supplementary Material

jead301_Supplementary_Data

## Data Availability

The majority of the data underlying this article can be shared upon reasonable request to the corresponding author, with the following exception. For data from NEDA, restrictions apply to the availability of these data, which were used under licence for the current study, and so are not publicly available.
