# Supplementary material for: Echocardiographic estimation of pulmonary artery wedge pressure: invasive derivation, validation, and prognostic association beyond diastolic dysfunction grading
Source: Eur Heart J Cardiovasc Imaging. 2023 Nov 9;25(4):498–509. doi: 10.1093/ehjci/jead301 (PMC10966330; doi:10.1093/ehjci/jead301)
Supplement: jead301_Supplementary_Data [file jead301_supplementary_data.docx]

**Supplemental material**

| **Table S1. Principal and/or contributing diagnoses after right heart catheterization in the derivation cohort** | |
| --- | --- |
| Heart failure with preserved ejection fraction | 17 (18.9) |
| Heart failure with reduced/mildly reduced ejection fraction | 10 (11.1) |
| Constrictive pericarditis | 9 (10) |
| Ischemic heart disease | 9 (10) |
| Pre-/post heart transplantation | 6 (6.7) |
| Restrictive cardiomyopathy | 5 (5.6) |
| Right ventricular failure | 5 (5.6) |
| Pulmonary fibrosis | 5 (5.6) |
| Normal | 4 (4.4) |
| Aortic stenosis | 4 (4.4) |
| Chronic thromboembolic pulmonary hypertension | 4 (4.4) |
| Systemic sclerosis | 4 (4.4) |
| Dilated cardiomyopathy | 3 (3.3) |
| Cardiac amyloid | 3 (3.3) |
| Hypertrophic cardiomyopathy | 3 (3.3) |
| Myocarditis | 2 (2.2) |
| Pulmonary hypertension | 2 (2.2) |
| Chronic obstructive pulmonary disease | 2 (2.2) |
| Arythmogenic cardiomyopathy | 1 (1.1) |
| Cirrhosis | 1 (1.1) |
| Idiopathic pulmonary arterial hypertension | 1 (1.1) |
| Respiratory insufficiency | 1 (1.1) |

| **Table S2. Principal and/or contributing diagnoses after right heart catheterization in the validation cohort** | |
| --- | --- |
| Heart failure with reduced/mildly reduced ejection fraction | 11 (20.8) |
| Systemic sclerosis | 6 (11.3) |
| Normal | 5 (9.4) |
| Heart failure with preserved ejection fraction | 5 (9.4) |
| Associated pulmonary arterial hypertension | 5 (9.4) |
| Chronic thromboembolic pulmonary hypertension | 5 (9.4) |
| Systemic lupus erythematosus /Mixed connective tissue disease | 4 (7.5) |
| Idiopathic pulmonary arterial hypertension | 3 (5.7) |
| Ischemic heart disease | 2 (3.8) |
| Chronic obstructive pulmonary disease | 2 (3.8) |
| Hypertrophic cardiomyopathy | 1 (1.9) |
| Constrictive pericarditis | 1 (1.9) |
| Aortic stenosis | 1 (1.9) |
| Ventricular septum defect | 1 (1.9) |
| Cardiac amyloid | 1 (1.9) |

|  |  |  |
| --- | --- | --- |
| **Table S3.** Selected baseline characteristics in males and females in the derivation and validation cohort | | |
|  | Males | Females |
| Age, years | 55±16 | 63±14 |
| ePAWP, mmHg | 16.1±5.9 | 13.4±4.3 |
| PAWP, mmHg | 16.6±8.3 | 12.8±8 |
| mPAP, mmHg | 28.2±11.9 | 27.4±11.5 |
| PVR, WU | 2.4±1.9 | 3.3±2.8 |
| LVEF | 50±21 | 60±15 |
| Abbreviations: LVEF: left ventricular ejection fraction; mPAP: mean pulmonary arterial pressure; PAWP: pulmonary arterial wedge pressure; PVR: pulmonary vascular resistance; WU: Wood units | | |

**Regression equation for estimation of pulmonary arterial wedge pressure (PAWP) using only left atrial volume indexed to body surface area (LAVi) and mitral early peak velocity (E):**

*ePAWP_E =_ 0.230 × LAVi + 10.177 × mitral E - 2.7,* in which ePAWP is given in mmHg, LAVi in ml/m^2^ and mitral E in m/s

**Agreement with invasive PAWP**: mean±SD difference 0.9±5.6 mmHg

**Area under the curve in detection of PAWP >15 mmHg:** 0.88 [0.79–0.98]

**Prognostic value:** ePAWP_E_ could be applied to 63,945 patients (5,152 events) and was associated with increased risk of cardiovascular death (C statistic 0.61 [0.60–0.62], unadjusted hazard ratio (HR) 1.06 [1.06 – 1.06] per mmHg; HR adjusted for age, sex and diastolic dysfunction 1.01 [1.01 – 1.02]).
